# Cdc42-Dependent Transfer of mir301 from Breast Cancer-Derived Extracellular Vesicles Regulates the Matrix Modulating Ability of Astrocytes at the Blood–Brain Barrier

**DOI:** 10.3390/ijms21113851

**Published:** 2020-05-28

**Authors:** Golnaz Morad, Cassandra C. Daisy, Hasan H. Otu, Towia A. Libermann, Simon T. Dillon, Marsha A. Moses

**Affiliations:** 1 Vascular Biology Program, Boston Children’s Hospital, Boston, MA 02115, USA; golnaz.morad@childrens.harvard.edu (G.M.); Cassandra.daisy@childrens.harvard.edu (C.C.D.); 2 Department of Surgery, Harvard Medical School, Boston, MA 02115, USA; 3 Graduate School of Arts and Sciences, Harvard University, Cambridge, MA 02138, USA; 4 Department of Electrical and Computer Engineering, University of Nebraska-Lincoln, Lincoln, NE 68588, USA; hotu2@unl.edu; 5 BIDMC Genomics, Proteomics, Bioinformatics and Systems Biology Center, Beth Israel Deaconess Medical Center, Boston, MA 02115, USA; tliberma@bidmc.harvard.edu (T.A.L.); sdillon1@bidmc.harvard.edu (S.T.D.); 6 Department of Medicine, Harvard Medical School, Boston, MA 02115, USA; 7 Department of Surgery, Boston Children’s Hospital, Boston, MA 02115, USA

**Keywords:** breast cancer, brain metastasis, extracellular vesicles, microRNA, blood–brain barrier

## Abstract

Breast cancer brain metastasis is a major clinical challenge and is associated with a dismal prognosis. Understanding the mechanisms underlying the early stages of brain metastasis can provide opportunities to develop efficient diagnostics and therapeutics for this significant clinical challenge. We have previously reported that breast cancer-derived extracellular vesicles (EVs) breach the blood–brain barrier (BBB) via transcytosis and can promote brain metastasis. Here, we elucidate the functional consequences of EV transport across the BBB. We demonstrate that brain metastasis-promoting EVs can be internalized by astrocytes and modulate the behavior of these cells to promote extracellular matrix remodeling in vivo. We have identified protein and miRNA signatures in these EVs that can lead to the interaction of EVs with astrocytes and, as such, have the potential to serve as targets for development of diagnostics and therapeutics for early detection and therapeutic intervention in breast cancer brain metastasis.

## 1. Introduction

Brain metastasis is a major complication of breast cancer and is associated with an extremely poor prognosis [1,2]. Elucidating the early mechanisms of brain metastasis development in breast cancer patients is therefore critical for the development of early diagnostics and effective therapeutics to improve the outcome of this disease.

During the early stages, brain metastases tend to grow along the brain vasculature, the blood–brain barrier (BBB) (also known as a vessel co-option pattern of growth) [3]. As such, the microenvironment surrounding the BBB serves as an initial niche for metastatic tumor cells [4]. Identifying the dynamic changes that occur in the microenvironment at the BBB prior to brain metastasis is essential to our understanding of the early mechanisms of brain metastasis.

It is widely acknowledged that tumor-derived extracellular vesicles (EVs) can promote tumor progression and metastasis. Once released into the circulation, these nanoscale vesicles can transfer their contents including proteins, lipids, DNA, and coding and non-coding RNA to cells in distant organs [5]. The resulting alterations in the behavior of these cells change the microenvironment in pre-metastatic organs in a manner that promotes future metastatic growth [6,7,8]. In the brain, the role of tumor-derived EVs in the progression of primary brain tumors has been studied extensively, but the current knowledge of their role in metastatic brain tumors is still limited [9]. EVs have demonstrated great promise as novel diagnostics and therapeutics for a variety of pathologies [10]. Understanding the role of breast cancer-derived EVs in brain metastasis can therefore provide opportunities for early detection and management of this disease. Our group and others have demonstrated that EVs derived from brain-seeking breast cancer cell lines (Br-EVs) can promote brain metastasis [11,12,13,14]. Our previous study has shown that these EVs can breach an intact BBB via a transcytosis process in vivo [11]. Importantly, we found that following their transcytosis, Br-EVs were taken up by astrocytes at the BBB, a process that is of potential mechanistic significance for the observed Br-EV-driven promotion of brain metastasis. In the current study, we focused on astrocytes as one of the major recipients of breast cancer-derived EVs in the brain [11] and sought to elucidate the mechanisms underlying the uptake of breast cancer-derived EVs by these cells and the associated functional consequences.

We demonstrate that astrocyte uptake of breast cancer-derived EVs relies on the Cdc42-dependent clathrin-independent carrier/GPI-AP-enriched compartment (CLIC/GEEC) endocytic pathway. Using quantitative proteomics analysis, we demonstrate the enrichment of a protein signature with the potential to interact with the CLIC/GEEC cargo. We next demonstrate that the uptake of Br-EVs by astrocytes changes the expression profile of astrocytes to prepare a tumor-supporting microenvironment at the BBB. We present data suggesting that at least one mechanism by which this process occurs is through alterations in the expression of extracellular matrix (ECM)-remodeling proteins by astrocytes. We further present data supportive of the role of miR-301a in EV-driven downregulation of TIMP-2.

## 2. Results

### 2.1. The Astrocyte Uptake of Breast Cancer-Derived EVs Is Mediated through the CLIC/GEEC Pathway

**Figure 1:**
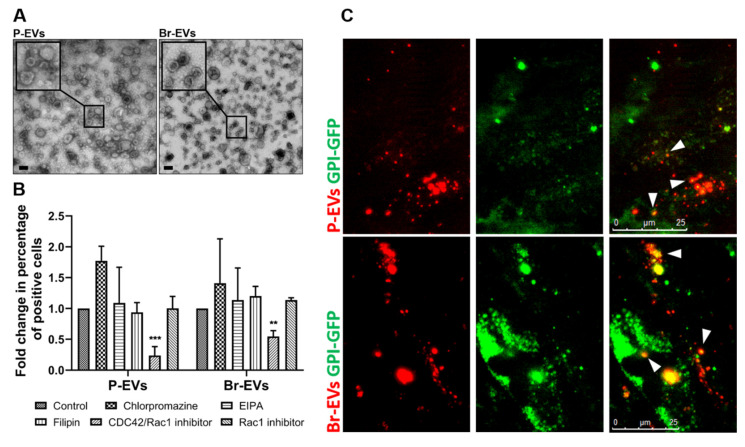
Astrocytes internalize breast cancer-derived extracellular vesicles (EVs) through the Cdc42-dependent clathrin-independent carrier/GPI-AP-enriched compartment (CLIC/GEEC) pathway. (**A**) Electron microscopy images of EVs isolated from parental and brain-seeking MDA-MB-231 breast cancer cells (P-EV and Br-EV, respectively). The square shows magnification of the selected area. (**B**) Flow cytometry quantification of TdTomato-labeled EV (TdTom-EV) uptake by astrocytes treated with chemical inhibitors of endocytosis pathways (mean ± SD; three independent experiments). Statistical analysis was performed using unpaired two-tailed Student’s *t*-test (** *p* ≤ 0.01; *** *p* ≤ 0.001). (**C**) Representative fluorescence microscopy images of the colocalization of TdTom-EVs (red) with GFP-fused glycosylphosphatidylinositol (GPI) (green) in astrocytes from three independent experiments. White arrows show the colocalization of the TdTomato and GFP signals. Scale bar, 25 µm.

Given the high incidence of brain metastasis in triple-negative breast cancer [1], we isolated EVs from the human triple-negative MDA-MB-231 cell line for which matched primary and brain-seeking variants are available (P-EVs and Br-EVs, respectively). The brain-seeking variant of these cells was established through six rounds of sequential passaging of parental cells in nude mice and was a gift from Dr. T. Yoneda, Indiana University [15]. Breast cancer-derived EVs were characterized according to the guidelines suggested by the International Society for Extracellular Vesicles [16] (Figure 1A and Figure S1). We have previously demonstrated astrocytes as one of the major recipients of Br-EVs in the brain in vivo [11], suggesting a prominent role for EV–astrocyte interactions in breast cancer brain metastasis.

To elucidate the mechanisms underlying the uptake of breast cancer-derived EVs by astrocytes, we first explored the possibility of the involvement of specific endocytosis mechanism(s) in this uptake. Endocytic pathways such as macropinocytosis, clathrin-dependent and caveolin-dependent endocytosis have been commonly reported to be involved in the uptake of EVs by different cell types [17]. We used chemical inhibitors of the different endocytosis pathways, including EIPA, chropromazine, and filipin to evaluate the effect of macropinocytosis, clathrin-dependent, and caveolin-dependent endocytosis, respectively. Interestingly, none of these inhibitors could inhibit the uptake of EVs (10^10^ particle/ well in a 12-well plate) following a 3 h incubation period, demonstrating that these common pathways were not involved in the uptake of EVs by astrocytes (Figure 1B). This finding was also in contrast to our previous findings demonstrating the involvement of macropinocytosis and clathrin-dependent endocytosis in the uptake of Br-EVs by brain endothelial cells [11] and emphasizes the cell-type dependency of EV uptake mechanisms.

We next explored the role of clathrin/caveolin-independent pathways in EV uptake, focusing on rac1 and Cdc42, two major players in this process [18]. We found that a Cdc42/Rac1 GTPase Inhibitor, ML141, significantly decreased the uptake of both types of EVs by astrocytes, whereas a specific Rac1 inhibitor, CAS 1177865-17-6, had no effect on their uptake, suggesting that Cdc42, but not Rac1, is involved in the uptake of breast cancer-derived EVs (Figure 1B).

Cdc42 is known to be involved in the endocytosis of glycosylphosphatidylinositol-anchored proteins (GPI-Aps) via the clathrin-independent carrier/GPI-AP-enriched compartments (CLIC/GEEC) pathway [19]. To evaluate whether EV uptake by astrocytes occurs through the Cdc42-dependent CLIC/GEEC pathway, we transfected astrocytes with a GFP-fused GPI construct [18]. High spatiotemporal resolution microscopy demonstrated the colocalization of TdTomato-labeled EVs with GPI (Figure 1C), confirming that the breast cancer-derived EVs share the endocytic pathway with GPI-APs.

Taken together, these findings demonstrated that the uptake of breast cancer-derived EVs by astrocytes is mediated through the non-canonical Cdc42-dependent CLIC/GEEC endocytosis pathway.

### 2.2. Br-EVs Are Enriched in Interacting Partners of the CLIC/GEEC Cargo

**Figure 2:**
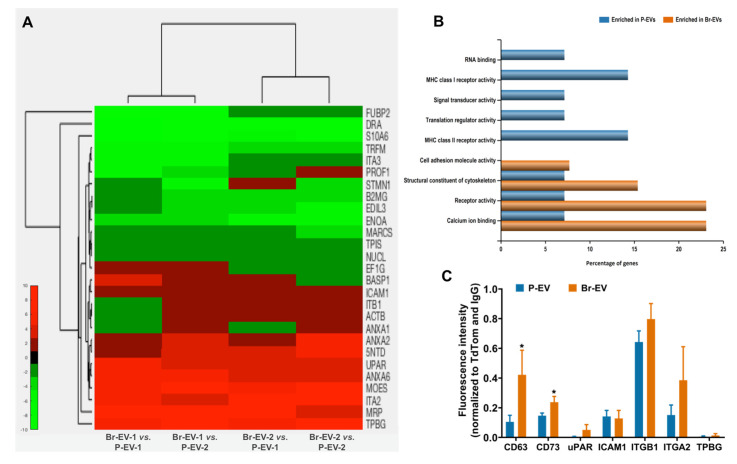
Br-EVs are enriched in interacting partners of the clathrin-independent carrier/GPI-AP-enriched compartments (CLIC/GEEC) cargo. (**A**) Heatmap visualization of quantitative proteomics analyses demonstrating the significantly differentially expressed proteins (*p* ≤ 0.05) in Br-EVs vs. P-EVs (red demonstrates upregulation in Br-EVs). (**B**) Functional enrichment analysis of proteins upregulated in P-EVs (blue) and Br-EVs (orange). (**C**) Quantification of surface localization of membrane-associated proteins upregulated in Br-EVs, CD63 serves as positive control (mean ± SD; three independent experiments). Statistical analysis was performed using unpaired two-tailed Student’s *t*-test (* *p* ≤ 0.05).

The endocytosis of EVs by different cell types is defined by the composition of surface proteins on EVs and their interaction with receptors and ligands on the cell membrane [20]. To identify the composition of proteins on breast cancer-derived EVs, we first performed quantitative mass spectrometry using the isobaric tag for relative and absolute quantitation (iTRAQ) technique on the two types of breast cancer-derived EVs. Among a total of 126 proteins detected with >95% confidence, 27 proteins were significantly (*p* ≤ 0.05) differentially expressed (14 upregulated, 13 downregulated) in Br-EVs compared to P-EVs (Figure 2A). Enrichment analysis using the FunRich software [21] demonstrated that the majority of these proteins belonged to receptor activity and cell adhesion categories (Figure 2B), supporting our hypothesis of their involvement in the specific interaction between breast cancer-derived EVs and astrocytes. We validated and quantified the surface localization of these proteins on P- and Br-EVs through staining of the intact EVs (Figure 2C). To this end, TdTomato P-EVs and Br-EVs (10^10^ particles in 100 µL PBS) were incubated with 5 µg/mL of fluorescent-conjugated antibodies for a 2 h incubation, following which EVs were washed through ultracentrifugation and fluorescence was measured using a plate reader.

Interestingly, a number of the surface proteins upregulated in Br-EVs have been previously identified as cargoes associated with the CLIC/GEEC pathway. While GPI-APs are the most studied cargo of the CLIC/GEEC pathway, a variety of other proteins, predominantly adhesion factors, have also been identified as the cargo of this endocytosis route. These include integrin β1, galectin 3, CD44, and CD98 [22,23,24]. Moreover, it has been shown that ICAM1 binding to integrins can induce nucleation and colocalization of integrin clusters and GPI-APs [24]. We demonstrated that Br-EVs were significantly enriched in Ecto-5′-nucleotidase (5NTD, also known as CD73), a well-known GPI-interacting protein, and also exhibited a higher expression trend for urokinase plasminogen activator receptor (uPAR, another GPI-interacting protein) [25,26], as well as integrin β1 and integrin α2. Both types of EVs had similar expression of ICAM1 on their surface (Figure 2C). CD63 is an EV-associated transmembrane protein that is known to be largely present on the surface of EVs and was used as a positive control in this experiment. Interestingly, we found that the level of CD63 was significantly higher in Br-EVs. Tetraspanins are known to associate with integrins on the membrane [27] and, therefore, elevated levels of CD63 on Br-EVs could be a potential explanation for the enrichment of integrins in these EVs. Together, these findings identify a combination of surface proteins upregulated in Br-EVs that have the potential to interact with GPI-AP clusters. These results are consistent with our previous findings, demonstrating a preferential uptake of Br-EVs compared to P-EVs by astrocytes in vitro [11]. Moreover, the enrichment of Br-EVs in surface proteins that can facilitate their internalization by astrocytes provides a potential explanation as to why Br-EVs but not P-EVs have the ability to promote brain metastasis, as previously reported by our group and others [11,14].

### 2.3. Br-EVs Decrease the Astrocyte Expression of TIMP-2

**Figure 3:**
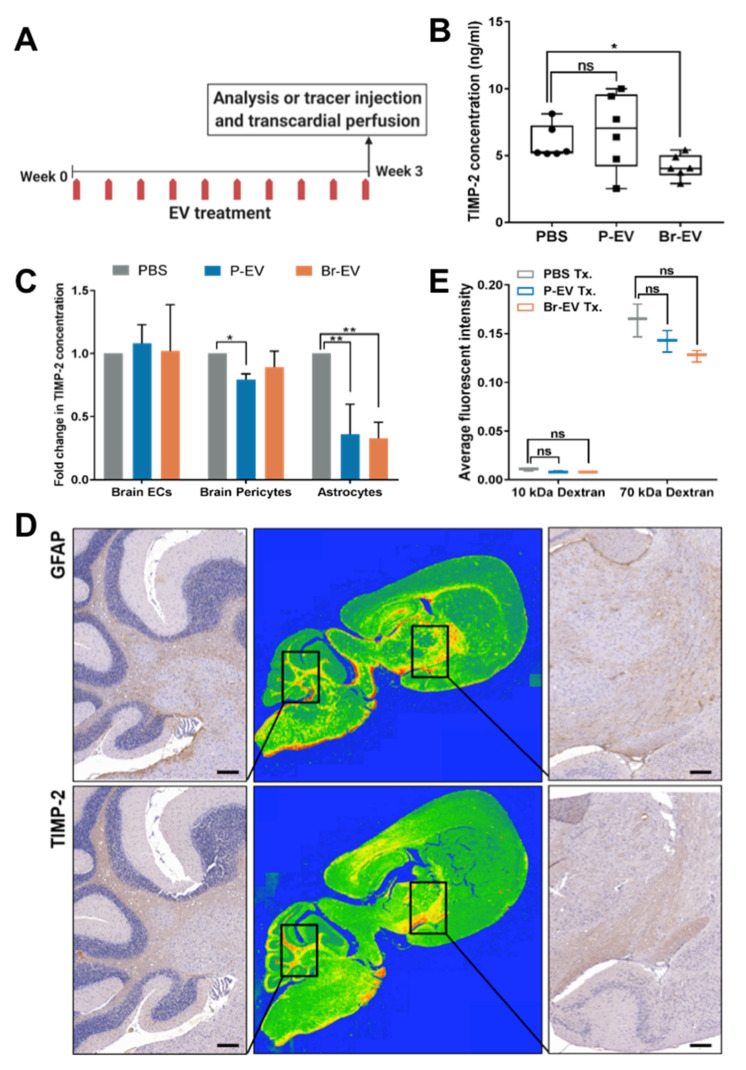
Br-EVs decrease the astrocyte expression of the tissue inhibitor of matrix metalloproteinases-2 (TIMP-2). (**A**) Schematic showing the EV functional study design. (**B**) Average concentration of TIMP-2 in brain tissue homogenates measured by a mouse TIMP-2 enzyme-linked immunosorbent assay (ELISA) (mean ± SD; n = six mice per group). Statistical analysis was performed using the Mann–Whitney test. (**C**) Average fold change in concentration of TIMP-2 in conditioned media of brain endothelial cells, pericytes and astrocytes treated with PBS, P-, and Br-EVs (mean ± SD; three independent experiments). Statistical analysis was performed using two-way ANOVA with Sidak’s multiple comparison tests. (**D**) Representative images of mouse brain sections immunostained with anti-GFAP (upper panels) and anti-TIMP-2 (lower panels), demonstrating colocalization of GFAP astrocyte marker and TIMP-2. Middle panels represent a colormap of areas of protein enrichment (three independent experiments). Scale bar, 200 µm. (**E**) Average fluorescence intensity in perfused brain tissue homogenates collected 45 min following injection of a combination of 10 KDa Alexa647 dextran and 70 KDa FITC dextran (mean ± SD; n = three mice per group). Statistical analysis was performed using the Mann–Whitney test. In all panels: ns, not significant; * *p* ≤ 0.05; ** *p* ≤ 0.01.

We next studied the functional consequences of EV uptake by astrocytes in vivo. We hypothesized that upon transcytosis through the brain endothelium, breast cancer-derived EVs can change the behavior of astrocytes to prepare a microenvironment supportive of tumor cell growth. It is widely acknowledged that matrix metalloproteinases (MMPs) and their endogenous inhibitors, the tissue inhibitors of MMPs (TIMPs), can contribute to tumor progression and metastasis [28,29,30,31]. Through modulating the ECM, these enzymes and their inhibitors can trigger different signaling pathways and promote the tumor-supporting microenvironment [5,32]. Several studies have demonstrated a prominent role for MMPs and TIMPs in preparation of a niche for tumor cell growth in the brain [33,34,35,36,37]. While these studies predominantly focus on tumor cell-derived MMPs and TIMPs, astrocyte-conditioned media were shown to modulate the tumor cell expression of MMPs. Moreover, astrocyte-derived MMP-2 and MMP-9 have also been shown to promote tumor cell invasion in breast cancer brain metastasis [38]. Accordingly, we postulated that Br-EVs can alter the expression of MMPs and TIMPs produced by astrocytes to facilitate brain metastasis. To address this hypothesis, we performed retro-orbital injections of P-EVs and Br-EVs (3 µg in 100 µL PBS per injection) in mice every two days for a total of 10 injections, following which the mice were sacrificed to analyze the brain tissue (Figure 3A). Retro-orbital injection is considered to be a superior route of administration for continuous injections by our institution’s IACUC and is commonly used for the injection of EVs into the circulation [6,39]. Using mouse-specific enzyme-linked immunosorbent assays (ELISA), we analyzed the expression of a number of MMPs and TIMPs that are known to be involved in ECM remodeling in brain tissue [40], including MMP-2, MMP-9, MMP-14, TIMP-1, and TIMP-2 in mouse brain tissue homogenates (Figure 3B, Figure S2A–D). Interestingly, we found that TIMP-2, the endogenous inhibitor of MMP activity [41], was exclusively and significantly decreased by brain metastasis-promoting Br-EVs, whereas this effect was not seen with P-EVs (Figure 3B).

We investigated whether astrocytes could be the source of the Br-EV-driven decrease in brain TIMP-2. We treated human BBB cells, endothelial cells, pericytes, and astrocytes, with P-EVs and Br-EVs in vitro (48 h incubation with 25 µg EVs) and evaluated TIMP-2 expression using a human-specific TIMP-2 ELISA. EV treatment did not affect the expression of TIMP-2 in brain endothelial cells (Figure 3C) but decreased the expression of TIMP-2 in astrocytes (Figure 3C). Moreover, Br-EVs were able to increase the migration of astrocytes, which is consistent with the observed decrease in TIMP-2 expression and a subsequent increase in ECM modulation (Figure S2E). This finding is consistent with our hypothesis that Br-EVs can change the behavior of astrocytes. Interestingly, both P-EVs and Br-EVs were able to induce TIMP-2 downregulation in vitro whereas, in vivo, this effect was exclusive to Br-EVs. These findings prompted the hypothesis that both EVs have the inherent ability to downregulate TIMP-2 in astrocytes, with Br-EVs being able to reach the astrocytes more efficiently in vivo. Our results with respect to the enrichment of Br-EVs in GPI-interacting proteins (Figure 2), along with our previous findings on the ability of Br-EVs to facilitate their transcytosis across the BBB [11], support this hypothesis.

To rule out the possibility that the decreased astrocyte TIMP-2 might be an indirect effect of Br-EVs acting through brain endothelial cells, we prepared conditioned media by treating brain endothelial cells with EVs or PBS. Astrocytes were incubated with the endothelial cell conditioned media for 48 h, followed by the media exchange and subsequent analysis of astrocyte-conditioned media. No difference in TIMP-2 levels was found in conditioned media from astrocytes that were incubated with PBS-, P-EV-, or Br-EV-treated endothelial cell-conditioned media (Figure S2F). In addition, we stained consecutive brain tissue sections for TIMP-2 and an astrocyte marker, GFAP, and found that areas that were rich in astrocytes also had a high expression of TIMP-2, further supporting astrocytes as the major source of TIMP-2 (Figure 3D).

We then evaluated whether the observed decrease in astrocyte TIMP-2 levels following Br-EV treatment in vivo were accompanied by alterations in the permeability of the BBB. To this end, we treated mice with P- or Br-EVs as described above (3 µg per injection for a total of 10 injections). Mice were subsequently injected with a combination of 10 KDa Dextran, Alexa Fluor™ 647 (300 µg), and 70 KDa FITC Dextran (2 mg) in 100 µL of PBS and brain tissues were collected for analysis after 45 min. No increase in permeability of brain endothelium to 10 and 70 KDa-dextran was observed following P- or Br-EV treatment (Figure 3E) in perfused brain tissues. This observation suggested that the BBB remained intact during this experiment, supporting the conclusion that the EV-induced decrease in TIMP-2 was a direct effect of transcytosed Br-EVs on astrocytes. Overall, these findings indicate that the transcytosis of Br-EVs and their subsequent uptake by astrocytes can have functional consequences, such as suppressed TIMP-2 expression, that can lead to the preparation of a microenvironment at the BBB suitable for the growth of metastases.

### 2.4. miR-301a-3p Transferred by Breast Cancer-Derived EVs Downregulate TIMP-2 in Astrocytes

To determine the EV factors driving the decrease in TIMP-2, we examined the role of EV miRNAs in this process. Previous reports have identified a number of miRNAs with the ability to target the 3′UTR of TIMP-2 mRNA in tumor cells, including miR-106a [42], miR-761 [43], and miR-301a [44]. Interestingly, in a whole miRNome analysis conducted by our group [45], treatment of brain endothelial cells by breast cancer-derived EVs increased the miR-301a-3p levels, suggesting the ability of breast cancer-derived EVs to transfer this miRNA into recipient cells. This observation prompted us to investigate the potential role of miR-301a-3p in the observed EV-driven downregulation of TIMP-2 in astrocytes.

**Figure 4:**
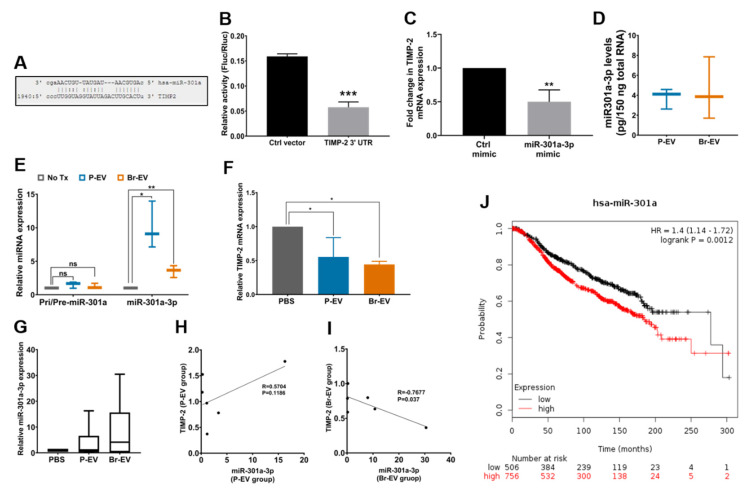
miR-301a-3p in breast cancer-derived EVs downregulates astrocyte TIMP-2. (**A**) Complementarity between the seeding sequence of miR-301a-3p and the 3′ UTR of TIMP-2. (**B**) Dual luciferase reporter assay to determine the physical interaction between miR-301a-3p and TIMP-2 3′ UTR (normalized to Renilla luciferase activity, mean ± SD; three independent experiments). Statistical analysis was performed using unpaired two-tailed Student’s *t*-test. (**C**) TIMP-2 mRNA levels in astrocytes following treatment with miR-301a-3p mimic (normalized to GAPDH, mean ± SD; three independent experiments). Statistical analysis was performed using unpaired two-tailed Student’s *t*-test. (**D**) Levels of miR-301a-3p in P-EVs and Br-EVs, measured against a standard curve created by miR-301a-3p mimic (mean ± SD; three independent experiments). Statistical analysis was performed using unpaired two-tailed Student’s *t*-test. (**E**) Level of primary/precursor or mature miR-301a in astrocytes following treatment with EVs (normalized to U6 expression, mean ± SD; three independent experiments). Statistical analysis was performed using two-way ANOVA with Sidak’s multiple comparison tests. (**F**) TIMP-2 level in astrocytes following treatment with EVs (normalized to GAPDH, mean ± SD; three independent experiments). Statistical analysis was performed using two-way ANOVA with Sidak’s multiple comparison tests. (**G**) Level of miR-301a-3p in brain tissue lysates (normalized to U6 levels, mean ± SD; n = six mice per group). Statistical analysis was performed using the Mann–Whitney test. (**H**,**I**) Correlation analysis between miR-301a-3p and TIMP-2 levels in brain tissue lysates in mice treated with P-EVs (**H**) and Br-EVs (**I**) (n = six mice per group). Correlation coefficient was measured using Pearson’s correlation analysis. (**J**) Kaplan–Meier curve demonstrating the association of miR-301a-3p levels with survival in breast cancer patients from the Molecular Taxonomy of Breast Cancer International Consortium (METABRIC) dataset [47]. In all panels: ns, not significant; * *p* ≤ 0.05; ** *p* ≤ 0.01; *** *p* ≤ 0.001.

Computational target prediction tools (www.miroRNA.org) demonstrated perfect complementarity between the miR-301a-3p seeding sequence and the TIMP-2 3′ UTR (Figure 4A). The ability of miRNAs to induce functional effects can differ based on how different cell types process EVs and their miRNA content [46]. To examine the ability of miR-301a-3p to physically interact with the 3′ UTR of TIMP-2 in astrocytes, we transfected the cells with a dual luciferase reporter vector of TIMP-2 3′ UTR or a control vector. Treatment with miR-301a-3p mimic (50 nM for 48 h) significantly decreased the luminescence activity in the TIMP-2 3′UTR-transfected cells, validating TIMP-2 as a target for miR-301a-3p (Figure 4B). Treatment of astrocytes with miR-301a-3p mimic also led to a decrease in endogenous TIMP-2 mRNA levels (Figure 4C), demonstrating the functionality of this miRNA in astrocytes.

To examine whether breast cancer-derived EVs carry this miRNA, we measured the miR-301a-3p levels in P- and Br-EVs and found that both types of EVs carried similar amounts of this miRNA (Figure 4D). To determine the ability of breast cancer-derived EVs to transfer this miRNA to astrocytes, we treated astrocytes with EVs under physiological culture conditions and measured the alterations in the levels of miR-301a-3p in astrocytes. Treatment of astrocytes with P- and Br-EVs (48 h incubation with 25 µg EVs) led to an increase in miR-301a-3p, demonstrating the transfer from EVs to astrocytes (Figure 4E). Furthermore, the levels of primary and precursor miR-301a were not changed following EV treatment, confirming that the observed increase in mature miRNA was not due to upregulation of endogenous miRNA in astrocytes and was a result of direct transfer from EVs. As expected, this increase in miR-301a-3p was associated with a downregulation of TIMP-2 mRNA (Figure 4F). Together, these findings demonstrated that breast cancer-derived EVs transfer miR-301a-3p to astrocytes, which can then directly target and downregulate TIMP-2 in these cells. We also evaluated the possibility of transferred miR-301a negatively affecting the continuous internalization of EVs by astrocytes; however, computational alignment tools suggested no complementarity between miR-301a-3p seeding sequence and the 3′ UTR of cdc42 (Needleman-Wunsch alignment [48], 0% identity).

To evaluate the ability of breast cancer-derived EVs to transfer this miRNA to the brain in vivo, we analyzed the level of miR-301a-3p in brain tissues collected from the in vivo experiment described above. It is important to note that the conserved and identical sequence of miR-301a-3p in mouse and human limited our ability to detect and analyze the direct transfer of miR-301a-3p by human breast cancer-derived EVs. Nevertheless, an increasing trend in the levels of miR-301a-3p was observed in mice that were treated with Br-EVs (Figure 4G). Importantly, the level of miR-301a-3p was significantly and negatively correlated with the level of TIMP-2 in Br-EV-treated mice, whereas this correlation was not observed in P-EV-treated mice (Figure 4H,I). These studies demonstrated a correlation between the level of miR-301a-3p and the observed downregulation of TIMP-2 in vivo. Given that Br-EV-driven downregulation of astrocyte TIMP-2 can occur prior to brain metastasis formation, miR-301a-3p has the potential to serve as a diagnostic marker for early stages of brain metastasis. Interestingly, analysis of 1262 patients in the Molecular Taxonomy of Breast Cancer International Consortium (METABRIC) [47] dataset demonstrated that higher levels of miR-301a-3p were significantly associated with decreased survival (www.kmplot.com, Figure 4J).

## 3. Discussion

In this study, we have elucidated the functional consequences of transcellular transport of breast cancer-derived EVs across the BBB, with a focus on the interaction of these EVs with astrocytes. We identified a series of mechanisms through which EVs are internalized by, and modulate, the behavior of astrocytes to promote a microenvironment supportive of metastatic growth.

We demonstrated that astrocytes internalize breast cancer-derived EVs through the specific Cdc42-dependent CLIC/GEEC pathway. To the best of our knowledge, this study is the first to report the uptake of EVs through this endocytosis pathway [16,49]. Interestingly, it has been shown that adeno-associated viruses can hijack the CLIC/GEEC pathway to gain entry into cells [50]. These findings are consistent with previous report of EVs using the virus entry machinery to enter cells [51]. Cells can internalize EVs through a variety of pathways including non-specific pathways (fusion, macropinocytosis) and receptor-mediated pathways. The uptake of EVs through receptor-mediated pathways is attributed to the interaction of EV surface proteins with ligands/receptors on the cell membrane [17]. However, the significant heterogeneity of EV populations suggests that multiple EV surface proteins are likely involved in the uptake of EVs by a particular cell type. Through a combination of proteomics analyses and localization studies, we identified a group of proteins, enriched on the surface of brain metastasis-promoting breast cancer-derived EVs. These proteins were recognized as interacting counterparts of several CLIC/GEEC pathway cargoes and therefore can play significant roles in the uptake of Br-EVs by astrocytes. Future studies incorporating these proteins into synthetic nanoparticles can evaluate the necessity and significance of each of these proteins for internalization by astrocytes. Furthermore, evaluating the presence of these proteins on single EVs through single-EV flow cytometry can provide information with regard to the distribution of these proteins among different subpopulations of EVs. Collectively, the identified protein signature can define a subpopulation of breast cancer-derived EVs that have the ability to interact with astrocytes and, in doing so, provide novel opportunities to address the longstanding challenge of dismantling the heterogeneity of EVs for identification of functional subpopulations.

Through in vitro and in vivo functional studies, we further demonstrated that Br-EVs can downregulate TIMP-2 in astrocytes. While the role of matrix metalloproteinases and their endogenous inhibitors in progression of metastasis has been studied extensively [31], this study is the first to demonstrate that tumor-derived EVs can initiate this process in the brain and provides insight into the early mechanisms involved in priming a niche prior to brain metastasis. We identified miR-301a-3p as the causal factor that can be transferred by breast cancer-derived EVs to astrocytes and downregulate TIMP-2.

Interestingly, our findings highlight differences between in vitro and in vivo behavior of EVs, which can have important mechanistic implications. First, we found that in contrast to our previous [11] and current studies suggesting a preferential in vitro uptake of Br-EVs by astrocytes, at a functional level, both P-EVs and Br-EVs carried similar amounts of miR-301a-3p and were able to induce similar effects on TIMP-2 in vitro. These differences are most likely due to the different duration of the uptake experiments compared to functional assays, during which cells had continuous and direct access to both types of EVs for a longer time allowing sufficient internalization of EVs to reach a functional threshold. Second, we found that despite the inherent ability of both types of EVs to downregulate TIMP-2 in astrocytes, this effect was only observed following treatment with Br-EVs but not P-EVs in vivo. The specificity in the function of Br-EVs in vivo can potentially be explained by a higher efficiency Br-EVs to reach the astrocytes in vivo. Importantly, and in support of this hypothesis, we have previously shown that Br-EVs, but not P-EVs, have the ability to modulate brain endothelial cells to facilitate their transcellular transport to reach astrocytes on the abluminal side [11]. More efficient internalization of Br-EVs by astrocytes could be another potential explanation; however, the limited resolution of the currently available technologies does not allow for reliable assessment of the preferential uptake of Br-EVs compared to P-EVs by astrocytes in vivo.

Taken together, our studies uncover novel mechanisms by which breast cancer-derived EVs prime the microenvironment in the brain following their transcytosis across the BBB. These mechanisms provide novel insights into the early events that occur prior to brain metastasis development from triple-negative breast cancer. It is important to note that the literature regarding triple-negative breast cancer brain metastasis is currently limited to the use of available matched primary and brain-metastatic cell lines. Development of transgenic models of spontaneous brain metastasis is critical for a better understanding of the mechanisms underlying brain metastasis.

The identified protein and miRNA signatures in this study have the potential to guide the development of diagnostics and therapeutics that would enable early interventions in triple-negative breast cancer brain metastasis. Future longitudinal preclinical studies and prospective clinical studies are required to validate the clinical implications of these findings.

## 4. Materials and Methods

### 4.1. Cell Lines and Cell Culture

Human breast cancer cell line MDA-MB-231 was purchased from American Type Culture Collection (ATCC^®^ HTB-26™, VA, USA). The brain-seeking variant of the breast cancer cell line MDA-MB-231 was a gift from Dr. T. Yoneda, Indiana University [15]. Primary human brain microvascular endothelial cells, astrocytes, and human brain vascular pericytes were purchased from Cell Systems Co. (Cat # ACBRI 376, Kirkland, WA, USA), Thermo Fisher Scientific Inc. (Cat # N7805100, Waltham, MA, USA), and ScienCell Research Laboratories (Cat # 1200, Carlsbad, CA, USA), respectively.

Breast cancer cells were cultured in Dulbecco’s Modified Eagle’s medium (DMEM, Cat # 11885084, Thermo Fisher Scientific Inc.) supplemented with 10% fetal bovine serum (FBS, Cat # S11150, Atlanta Biologicals^TM^, Atlanta, GA, USA) and 1% Penicillin-Streptomycin (10,000 U/mL) (Cat # 15140148, Thermo Fisher Scientific Inc.). For extracellular vesicle (EV) isolation, breast cancer cells were cultured in Advanced DMEM supplemented with 10% EV-depleted FBS. The EV-depleted FBS-containing medium was prepared as described previously [11,52]. Human brain endothelial cells were cultured with the endothelial cell growth medium (EGM™-2MV, Cat # CC-3202, Lonza Inc., Rockland, ME, USA). Human astrocytes and brain pericytes were cultured according to the manufacturer’s instructions. All cells were maintained in a 37 °C humidified incubator with 5% CO_2_. All cultures were routinely monitored for mycoplasma contamination using the MycoAlert™ PLUS Mycoplasma Detection Kit (Cat # LT07-710, Lonza Inc.).

### 4.2. EV Isolation and Characterization

EVs were isolated from 24–48 h conditioned media from breast cancer cell cultures with >95% cell viability, using a sequential centrifugation technique [11]. Briefly, conditioned media were centrifuged at 400× *g* for 10 min, 2000× *g* for 15 min, and 15,000× *g* for 30 min at 4 °C (Sorvall^®^ RC-5B centrifuge, Thermo Fisher Scientific Inc.) followed by ultracentrifugation at 100,000× *g* for 90 min at 4 °C (Optima XE-90 Ultracentrifuge, Beckman Coulter Life Sciences). EV pellets were washed at 100,000× *g* for another 90 min and were resuspended in PBS.

EV preparations were characterized according to the guidelines of the International Society for Extracellular Vesicles [16] and as described previously by us [11]. EV size and concentration was measured by nanoparticle tracking analysis (NanoSight NS300, Malvern Instruments, UK). EV markers were evaluated by western blot and the shape of the EVs was evaluated by electron microscopy [11].

To isolate TdTomato-labeled EVs, we transduced breast cancer cells with a lentiviral vector to express palmitoylated TdTomato (PalmtdTomato) [53]. The DNA construct was a gift from Dr. X. Breakefield, Massachusetts General Hospital. The fluorescence label of the isolated EVs were evaluated by fluorescent microscopy and plate reader (SpectraMax M2 plate reader, Molecular Devices LLC, San Joses, CA, USA).

### 4.3. In Vitro EV Uptake Studies

To evaluate the uptake of EVs by astrocytes for endocytosis inhibition studies, astrocytes were treated with chlorpromazine hydrochloride (Cat # C8138, 20 µM, Millipore Sigma, Woodlands, TX, USA), 5-(N-Ethyl-N-isopropyl) amiloride (EIPA) (50 µM, Tocris, Cat # 3378), filipin III (10 µM, Millipore Sigma, Cat # F4767), CDC42/Rac1 inhibitor, ML141 (100 µM, Millipore Sigma, Cat # 217708), rac1 inhibitor, or CAS 1177865-17-6 (10 µM, Millipore Sigma, Cat # 553502) for 30 min. Subsequently, TdTom-Br-EVs (10^10^ particle/well in a 12-well plate) were incubated with astrocytes for 3 h, following which the cells were washed and EV uptake was measured by flow cytometry using a BD FACSCalibur flow cytometer (BD Biosciences, San Jose, CA, USA).

To evaluate the colocalization of EVs with GPI-APs in astrocytes, cells were initially transfected with GFP-GPI plasmid using Lipofectamine 3000 reagent (Thermo Fisher Scientific). GFP-GPI WT plasmid was a gift from Dr. A.K. Hadjantonakis (Addgene plasmid # 32601; http://n2t.net/addgene:32601; RRID:Addgene_32601, Addgene, Watertown, MA, USA) [19]. Transfected cells were cultured on glass-bottom microslides (Ibidi, Cat # 80827) and were incubated with TdTom-Br-EVs (8 × 10^9^ particles/well) for 30 min. Subsequently, cells were washed 4 times with PBS and fixed with 4% formaldehyde for 10 min. Epifluorescence microscopy was performed on a Leica microscope coupled to high-resolution objectives and a Hamamatsu Orca CCD (Japan).

### 4.4. In Vitro EV Functional Studies

To evaluate the direct effect of EVs on the expression profile of BBB cells, primary human brain ECs, astrocytes, and pericytes cultured in 12-well plates were treated with P- or Br-EVs for 48 h (25 µg EVs per treatment). Conditioned media were collected for downstream analyses. The amount of TIMP-2 was measured in conditioned media using a human TIMP-2 ELISA (R&D Systems Inc. Cat # DTM200) according to the manufacturer’s instructions.

For astrocyte migration studies following continuous EV treatment, astrocytes were trypsinized and were plated in Transwell filters (8 μm pores, Costar Transwell Assay; Corning Inc., Corning NY) in astrocyte serum-free media (2 × 10^4^ cells in 100 μL media per filter). Filters were placed in 24-well plates containing 600 μL of complete astrocyte media. After 16 h, cells were fixed and stained with DAPI. Cells attached to the top of the filter were removed. Membranes were separated from the filters and were mounted on glass slides. The number of cells on the bottom of the filters were counted using a Zeiss Axiocam fluorescent microscope at 200× magnification (4 fields/filter, n = 2 filters per treatment).

To assess any indirect effects of EVs on astrocytes, brain ECs were initially treated with P- or Br-EVs for 3 consecutive days days (5 µg EVs per treatment), as previously described [11]. After treatment, EC-conditioned media were collected. Astrocytes cultured in 6-well plates were incubated with EC-conditioned media for 48 h, following which the cells were serum starved overnight and astrocyte-conditioned media were collected for analysis. TIMP-2 levels were measured using a human TIMP-2 ELISA (Cat # DTM200, R&D Systems Inc., Minneapolis, MN, USA) according to the manufacturer’s instructions.

### 4.5. Validation of EV Surface Proteins

TdTomato P-EVs and Br-EVs (10^10^ particles in 100 µL PBS) were incubated with 5 µg/mL of fluorescent-conjugated antibodies: FITC anti-human CD73 antibody (Cat # 344015, BioLegend, San Diego, CA, USA), FITC anti-human uPAR antibody (Sino Biological, Cat # 10925-MM09-F), Alexa Flour^®^ 488 anti-human ICAM1/CD54 antibody (BioLegend, Cat # 322713), Alexa Flour^®^ 488 anti-human CD29 antibody (BioLegend, Cat # 303015), FITC anti-human CD49b antibody (BioLegend, Cat # 359305), Alexa Flour^®^ 488 anti-human CD63 antibody (BioLegend, Cat # 353037), Alexa Flour^®^ 488 mouse IgG1, κ isotype control (BioLegend, Cat # 400132), and FITC mouse IgG1, κ isotype control (BioLegend, Cat # 400107). Following a 2 h incubation at room temperature, EVs were washed through ultracentrifugation to remove any free antibodies. Pellets were resuspended in PBS and fluorescence intensity was measured using a SpectraMax M2 plate reader. The fluorescence intensity of FITC or Alexa Flour^®^ 488 was normalized to that of TdTomato for both antibodies and isotype controls. The normalized measurements of antibodies were then subtracted from those of isotype controls.

### 4.6. Proteomics Analysis

Quantitative proteomics analysis was performed using the isobaric tags for relative and absolute quantitation (iTRAQ) technique as we have described previously [54]. For protein identification, the peak list was searched against the Swiss-Prot database including all human proteins. Both detection and differential expression analyses were carried out using the ProteinPilot v3.0 software (AB SCIEX). An unbiased ProtScore of >1.3, which corresponds to 95% confidence in detection (*p* < 0.05) was used for analysis. Significantly differentially expressed proteins between two samples were identified based on the ratio of the protein expression levels in the two samples (*p* < 0.05). Hierarchical clustering of samples and features was done using the unweighted pair group method with arithmetic mean (UPGMA) method, with Pearson’s correlation as the distance measure [55]. The expression data matrix was row-normalized prior to the application of average linkage clustering. Functional enrichment analysis of the proteins that were enriched in Br-EVs was performed using the FunRich software, v3.1.3 [21].

### 4.7. In Vivo Experiments

All animal experiments were conducted in accordance with the Institutional Animal Care and Use Committee (IACUC) guidelines of the Boston Children’s Hospital, Boston, MA (Identification Code 18-05-3740R and Date of Approval 10/9/2019).

Six to eight-week-old female Nu/Nu nude mice were purchased from Massachusetts General Hospital. Following 4–7 days of acclimation, mice were randomly divided into 3 groups and received retro-orbital injections of PBS, P-EVs or Br-EVs (3 µg in 100 µL PBS per injection). Injections were repeated every two days for a total of 10 injections, following which the mice were sacrificed and brain tissue was collected for analysis. For each brain, the left hemisphere was snap-frozen in liquid nitrogen. Tissue homogenates were prepared as described above. The expression of MMPs and TIMPs was evaluated using ELISAs for MMP-2 (R&D Systems Inc. Cat # MMP200), MMP-9 (R&D Systems Inc. Cat # MMPT90), MMP-14 (Cat # LS-F7353, Lifespan Biosciences Inc., Seattle, WA, USA), TIMP-1 (R&D Systems Inc. Cat # MTM100), and TIMP-2 (Abcam, Cat # ab100746). All kits were mouse specific, except for the MMP-2 ELISA kit that could recognize both human and mouse MMP-2. All assays were conducted according to the manufacturers’ instructions. The right hemisphere was fixed in 10% formalin. Formalin-fixed and paraffin-embedded tissue sections were analyzed using anti-TIMP-2 antibody (1:1000, Cat # GB11523, Servicebio, Wuhan, China) and anti-GFAP antibody (1:1000, Servicebio, Cat # GB11096). Immunohistochemistry was conducted as previously described [11].

To evaluate the integrity of the BBB during this experiment, the experiment was conducted as described above. Following the 3 week EV treatment, at the time of sacrifice, mice received a retro-orbital injection of a combination of 10 KDa Dextran, Alexa Fluor™ 647 (300 µg), and 70 KDa FITC Dextran (2 mg), in 100 µL of PBS. After 45 min, perfusion was performed with 25 mL of PBS. Collected brains were snap-frozen in liquid nitrogen for tissue lysate preparation. Brain tissue lysates were prepared in tissue protein extraction reagent (T-PER™) supplemented with Halt™ protease inhibitor cocktail (Thermo Scientific) using a 0.9–2.0 mm stainless steel bead blend (Next Advance Inc.). Fluorescence intensity was measured using a SpectraMax M2 plate reader and was normalized to tissue weight. Homogenates from brain tissue of non-treated mice were used to measure the tissue autofluorescence.

### 4.8. miRNA Target Validation

For target validation, astrocytes were transfected with dual luciferase reporters (TIMP-2 and control clones, Cat # HmiT018093-MT06, and CmiT000001-MT06, respectively, GeneCopoeia™, Rockville, MD, USA) according to the manufacturer’s instructions. Following 48 h, cells were transfected with miRNA-301a-3p mimics (50 nM miRIDIAN, Dharmacon Inc., Lafayette, CO, USA) using the Dharmafect 4 transfection reagent. Luciferase assays were conducted 48 h after mimic treatment, using the Luc-Pair™ Duo-Luciferase HS Assay Kit (GeneCopoeia™), according to the manufacturer’s instructions. For functional evaluation of miR-301a-3p, astrocytes were treated with 50 nM miRNA-301a-3p mimics for 48 h, after which RNA was isolated for analysis.

### 4.9. RNA Isolation and Analysis

RNA isolation from EV samples, astrocytes, and brain tissue was conducted using the miRNeasy kit (Qiagen, Germantown, MD, USA), according to the manufacturer’s instructions. Brain tissues were homogenized in Qiazol reagent using the stainless steel bead blend, as described previously. For analysis of TIMP-2 mRNA expression, mature miRNA expression and miRNA precursor analyses, we used the SuperScript™ VILO™ cDNA Synthesis Kit and the SYBR™ Green PCR Master Mix (ThermoFisher Scientific), miRCURY LNA RT Kit and SYBR Green PCR kit (Qiagen), and the miScript II RT kit and SYBR Green PCR kit (Qiagen), respectively. The following primers were used for these studies: PrimePCR™ SYBR^®^ Green Assay: TIMP2, Human (Bio-Rad, assay ID qHsaCID0022953); miRCury LNA miRNA PCR assays (U6 snRNA-hsa, hsa-miR-301a-3p, hsa-miR-301b-3p) and Hs_miR-301a_1_PR miScript precursor assay, and Hs_miR-301a_1 and Hs_RNU6-2_11 miScript primer assays (Qiagen).

### 4.10. Statistical Analyses

Statistical analyses were performed using the GraphPad Prism software, v8.3.0 (Graphpad, San Diego, CA, USA). All quantified data are presented as the mean ± SD from three independent experiments. Statistical significance was considered at *p* values lower than 0.05. *p* values were shown as * *p* ≤ 0.05; ** *p* ≤ 0.01; *** *p* ≤ 0.001; **** *p* ≤ 0.0001. The methods of statistical analyses have been indicated in figure legends. All comparisons between two experimental groups were performed by unpaired two-tailed Student’s *t*-test. Comparisons between more than 2 groups were performed by one-way ANOVA with Tukey’s correction for multiple comparisons. Groups of data involving more than one variable were analyzed by two-way ANOVA with Sidak’s correction for multiple comparisons. For in vivo experiments, the minimum number of animals required to conduct statistical analysis were included in the study and were randomly assigned into experimental groups. All in vivo experiments were evaluated using the Mann–Whitney test. The correlation between miR-301a-3p and TIMP-2 levels was evaluated via Pearson’s correlation test.

## Supplementary Materials

Supplementary materials can be found at https://www.mdpi.com/1422-0067/21/11/3851/s1.
