# Supplementary material for: Cdc42-Dependent Transfer of mir301 from Breast Cancer-Derived Extracellular Vesicles Regulates the Matrix Modulating Ability of Astrocytes at the Blood–Brain Barrier
Source: Int J Mol Sci. 2020 May 28;21(11):3851. doi: 10.3390/ijms21113851 (PMC7311991; doi:10.3390/ijms21113851)
Supplement: Supplementary file 1 [file ijms-21-03851-s001.zip › Morad et al._Supplementary figures.pdf]

Supplemental Figures for:

Cdc42-Dependent Transfer of mir301 from Breast Cancer-Derived Extracellular Vesicles Regulates the Matrix Modulating Ability of Astrocytes at the Blood–Brain Barrier

Golnaz Morad[1,2,3], Cassandra C. Daisy[1], Hasan H. Otu[4], Towia A. Libermann[5,6], Simon T. Dillon[5,6], Marsha A. Moses *[1,2,7]

[1] Vascular Biology Program, Boston Children's Hospital, Boston, MA, 02115, USA;
[2] Department of Surgery, Harvard Medical School, Boston, MA, 02115, USA;
[3]Graduate School of Arts and Sciences, Harvard University, Cambridge, MA, 02138, USA;
[4]Department of Electrical and Computer Engineering, University of Nebraska-Lincoln, Lincoln, NE, 68588, USA;
[5]BIDMC Genomics, Proteomics, Bioinformatics and Systems Biology Center, Beth Israel Deaconess Medical Center, Boston, MA, 02115, USA;
[6]Department of Medicine, Harvard Medical School, Boston, MA, 02115, USA;
[7]Department of Surgery, Boston Children's Hospital, Boston, MA, 02115, USA.

*To whom correspondence should be addressed:
Marsha A. Moses, Ph.D.
Vascular Biology Program
Boston Children's Hospital
The Karp Family Research Building
300 Longwood Avenue, Boston MA 02115
Phone (617) 919-2207
Fax (617) 730-0231
Email: marsha.moses@childrens.harvard.edu

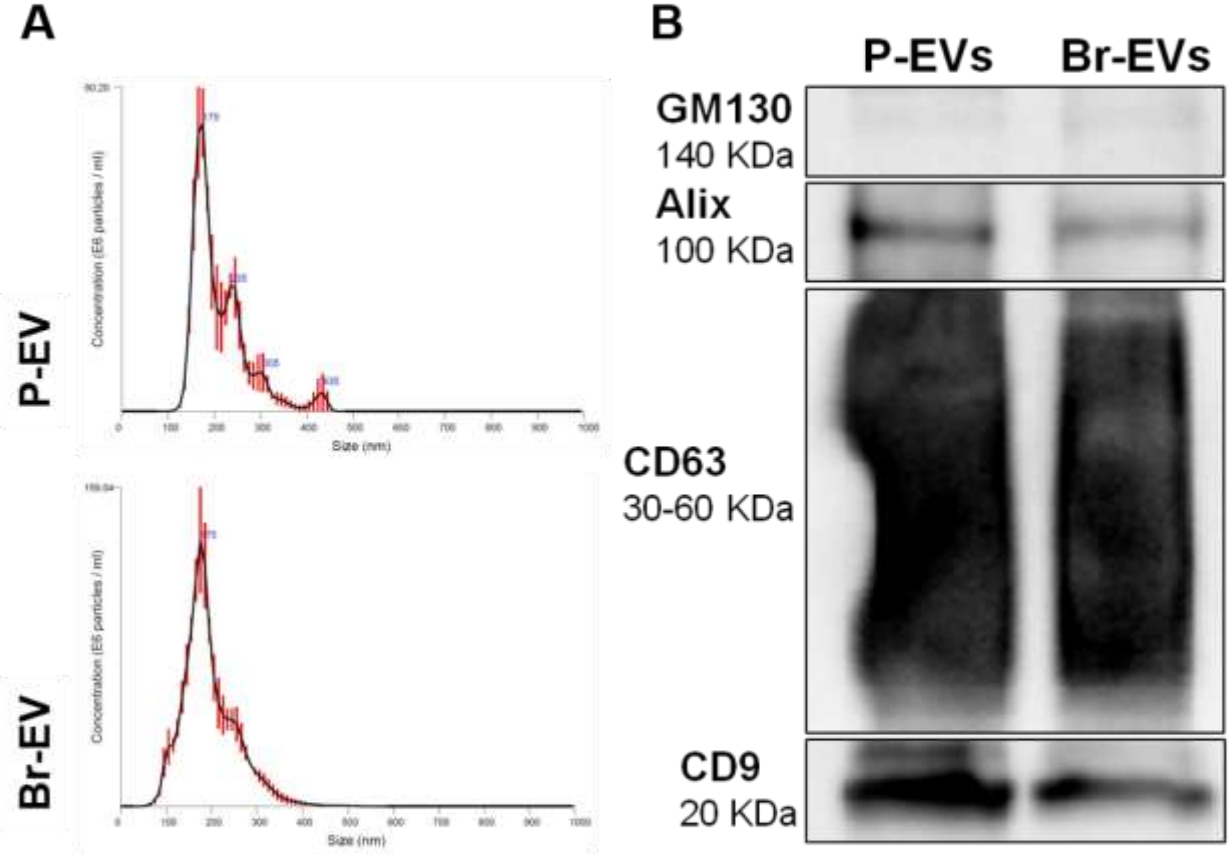

Figure S1. Characterization of breast cancer-derived EVs. (A) Nanoparticle tracking analysis of the size of P-EVs and Br-EVs. (B) Representative western blot images of EV markers CD9, CD63, Alix, and the golgi marker, GM130.

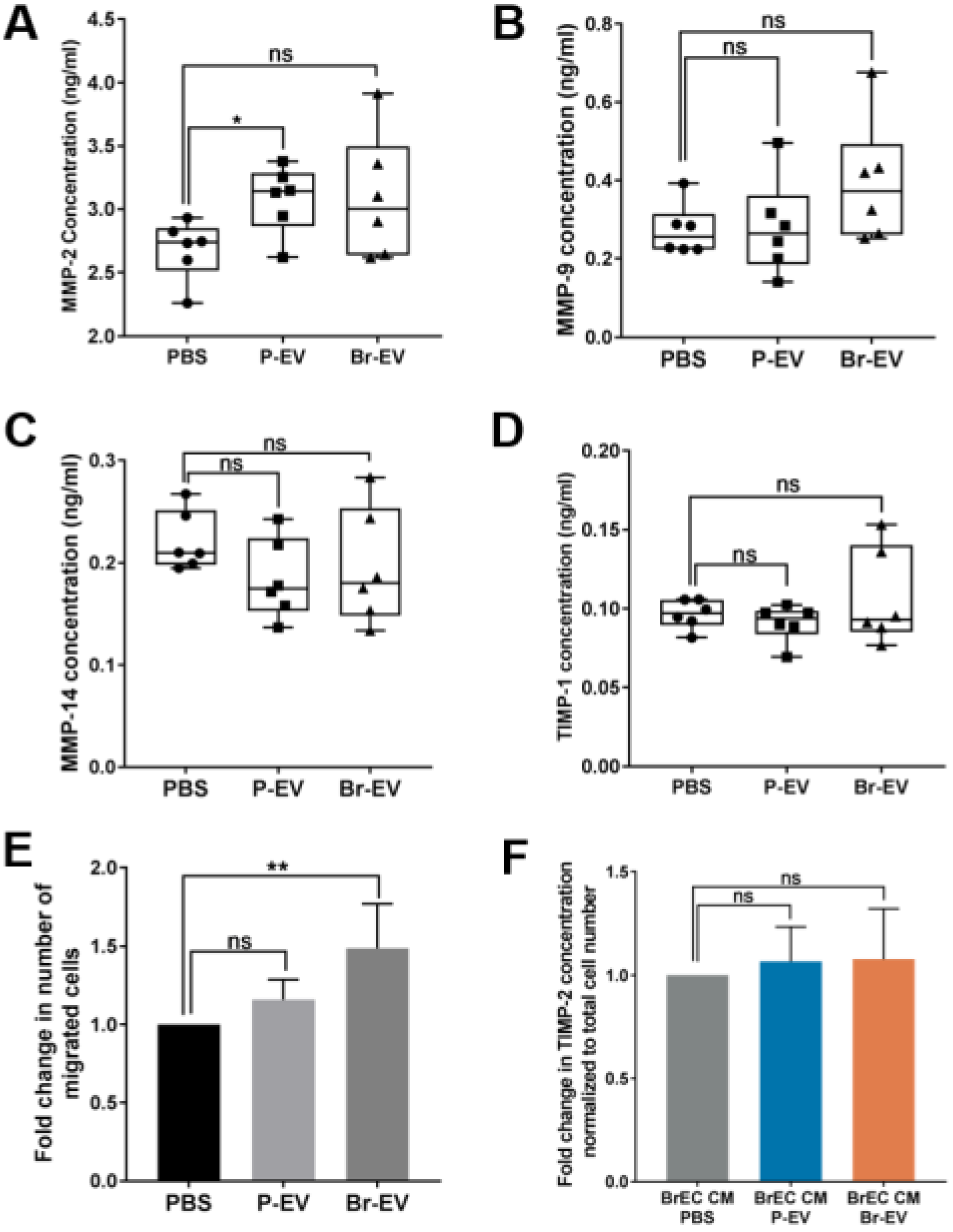

Figure S2. Effect of breast cancer-derived EVs on matrix metalloproteinases and their endogenous inhibitors. Average concentration of (**A**) MMP-2, (**B**) MMP-9, (**C**) MMP-14, and (**D**) TIMP-1 in brain tissue homogenates measured by ELISA (mean ± SD; n= 6 mice per group). Statistical analysis was performed using Mann-Whitney test. (**E**) Fold change in the number of migrated astrocytes in a transwell migration assay following pre-treatment with PBS, P- or Br-EVs (mean ± SD; 3 independent experiments). Statistical analysis was performed using one-way ANOVA with Tukey's test for multiple comparison. (**F**) Fold change in the concentration of TIMP-2 in astrocyte conditioned media following treatment with conditioned media from PBS-, P-EV, and Br-EV-treated endothelial cells (mean ± SD; 3 independent experiments). Statistical analysis was performed using one-way ANOVA. In all panels, ns, not significant; * $p \leq 0.05$; ** $p \leq 0.01$; *** $p \leq 0.001$.
