# Supplementary figures and images for: Cdc42-Dependent Transfer of mir301 from Breast Cancer-Derived Extracellular Vesicles Regulates the Matrix Modulating Ability of Astrocytes at the Blood–Brain Barrier
Source: Int J Mol Sci. 2020 May 28;21(11):3851. doi: 10.3390/ijms21113851 (PMC7311991; doi:10.3390/ijms21113851)

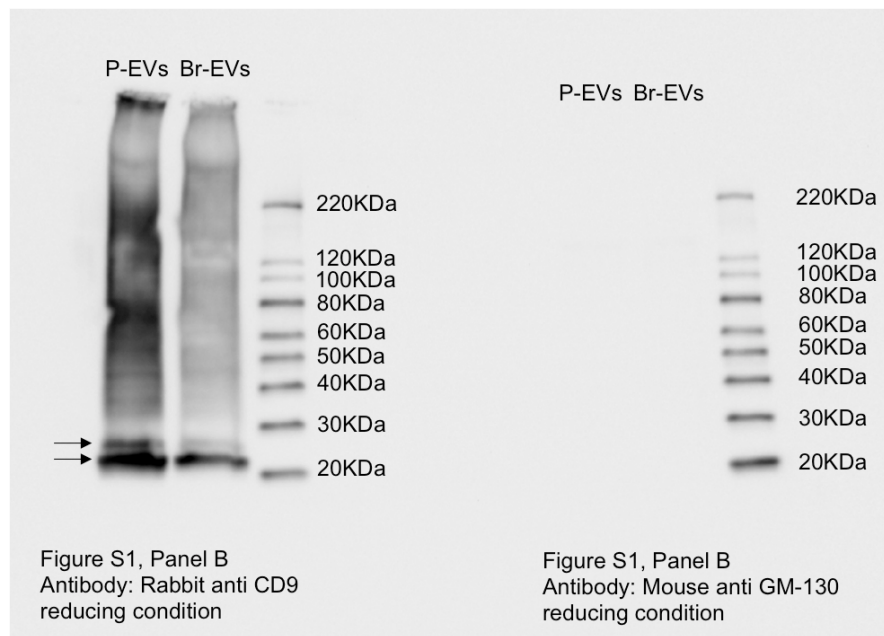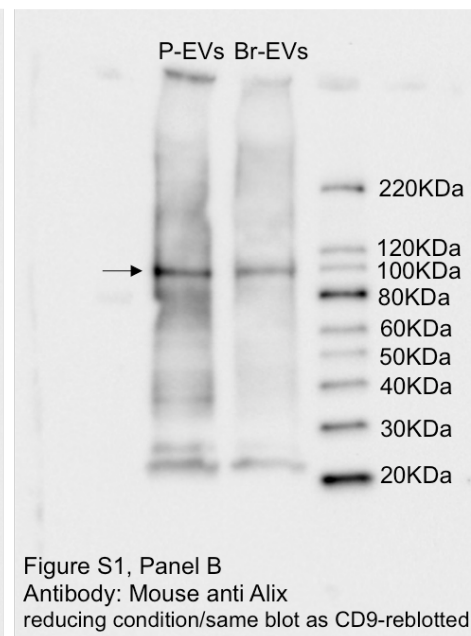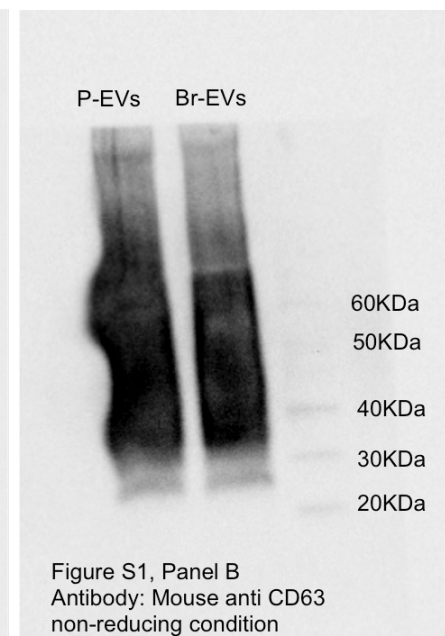

Supplement: Supplementary file 1 [file ijms-21-03851-s001.zip › Morad et al._Uncut blots.pdf]
